# Revised international staging system allocation in the ICARIA‐MM study: Practical challenges and impact on outcome

**DOI:** 10.1002/jha2.328

**Published:** 2021-11-07

**Authors:** Paul G. Richardson, Aurore Perrot, Hiroyuki Takamatsu, ICARIA‐MM study group

**Affiliations:** ^1^ Jerome Lipper Multiple Myeloma Center Department of Medical Oncology Harvard Medical School Dana‐Farber Cancer Institute Boston Massachusetts USA; ^2^ CHU de Toulouse IUCT‐O UPS Service d'Hématologie Université de Toulouse Toulouse France; ^3^ Department of Hematology Faculty of Medicine Pharmaceutical and Health Sciences/Faculty of Transdisciplinary Sciences Institute of Medical Institute of Transdisciplinary Sciences Kanazawa University Kanazawa Japan

## Dear Editor

The aim of the ICARIA‐MM Phase 3 study (ClinicalTrials.gov, number NCT02990338) was to determine the progression‐free survival benefit of isatuximab plus pomalidomide and dexamethasone compared with pomalidomide and dexamethasone alone in patients with relapsed and refractory multiple myeloma [1]. Results from this study demonstrated that the addition of isatuximab to pomalidomide and dexamethasone provides a significant benefit for progression‐free survival over pomalidomide and dexamethasone alone. Additionally, results showed a positive treatment effect in all subgroups, including revised international staging system (R‐ISS) stage at study entry [1].

The R‐ISS, as defined by the international myeloma working group (IMWG), combines the original ISS (beta‐2 microglobulin and albumin) with chromosomal abnormalities (del[17p] and/or t[4;14] and/or t[14;16]; determined by fluorescence in situ hybridization) and lactate dehydrogenase (LDH) results [2]. Like the ISS, the R‐ISS is based on three stages; patients with beta‐2 microglobulin <3.5 mg/L and albumin ≥3.5 g/dL, standard‐risk for cytogenetics abnormalities and normal LDH (≤upper limit of normal) are allocated as stage I; patients with beta‐2 microglobulin of ≥5.5 mg/L and either high‐risk for cytogenetics abnormalities or high LDH (>upper limit of normal) are allocated to stage III; and patients who do not meet criteria for stage I or stage III are allocated to stage II.

In the original analysis of the ICARIA‐MM study results, the interpretation of the IMWG criteria for R‐ISS allocated patients with unknown cytogenetic abnormalities or missing beta‐2 microglobulin at baseline as R‐ISS stage I or stage II (Table 1) [1]. R‐ISS was allocated based on several parameters measured at baseline, including beta‐2 microglobulin, albumin, LDH and cytogenetic abnormalities (characterized by central laboratory fluorescence in situ hybridization testing of purified CD138+ plasma cells from baseline bone marrow aspirate). Among patients for whom the cytogenetic abnormalities status was unknown, but other variables were available and allowed the R‐ISS status to be determined, 19 patients were allocated as R‐ISS stage I and nine patients as R‐ISS stage II. In addition, six patients with missing beta‐2 microglobulin were allocated as R‐ISS stage II, following the R‐ISS definition that suggests this stage when a patient does not meet criteria to be allocated as stage I or stage III (Table 1).

**TABLE 1:** R‐ISS stage at study entry according to beta‐2 microglobulin, albumin, LDH and cytogenetic abnormalities results measured at baseline (original allocation used in ICARIA‐MM), intent‐to‐treat population

Beta‐2 microglobulin (mg/L)	Albumin (g/dL)	LDH	Cytogenetic abnormalities (FISH)	R‐ISS	Number of patients
<3.5	≥3.5	≤ULN	High‐risk	Stage II	11
			Standard‐risk	Stage I	51
			Unknown		19
			High‐risk	Stage II	15
			Standard‐risk		32
			Unknown		14
≥3.5–<5.5			High‐risk	Stage II	14
			Standard‐risk		51
			Unknown		17
≥5.5			High‐risk	Stage III	20
		>ULN	Standard‐risk		15
			Unknown		5
		≤ULN	Standard‐risk	Stage II	28
			Unknown		9
Missing			High‐risk	Stage II	0
			Standard‐risk		4
			Unknown		2

Abbreviations: FISH, fluorescence in situ Hybridization; LDH, lactate dehydrogenase; R‐ISS, revised international staging system; ULN, upper limit of normal.

A more conservative way of allocating the R‐ISS status would be to separate the patients with unknown cytogenetic abnormalities and missing beta‐2 microglobulin at baseline into a fourth category named 'unclassified'. In the ICARIA‐MM study, a total of 34 patients (19 R‐ISS stage I and 15 R‐ISS stage II according to the original allocation) would have been allocated to 'unclassified' (Table 2).

**TABLE 2:** R‐ISS stage at study entry according to the original allocation used in ICARIA‐MM and to the alternative allocation method, intent‐to‐treat population

	Isa‐Pd (*n* = 154)	Pd (*n* = 153)	All (*N* = 307)
R‐ISS stage at study entry, original allocation, *n* (%)			
Stage I	39 (25.3)	31 (20.3)	70 (22.8)
Stage II	99 (64.3)	98 (64.1)	197 (64.2)
Stage III	16 (10.4)	24 (15.7)	40 (13.0)
Unknown	0	0	0
R‐ISS stage at study entry, alternative allocation, *n* (%)			
Stage I	31 (20.1)	20 (13.1)	51 (16.6)
Stage II	91 (69.1)	91 (59.5)	182 (59.3)
Stage III	16 (10.4)	24 (15.7)	40 (13.0)
Unclassified	16 (10.4)	18 (11.8)	34 (11.1)

Abbreviations: d, dexamethasone; Isa, isatuximab; P, pomalidomide; R‐ISS, revised international staging system.

To further investigate if this alternative R‐ISS allocation approach would lead to different conclusions about the ICARIA‐MM study, the authors conducted a post hoc analysis to assess the progression‐free survival benefit using the alternative R‐ISS staging allocation in which the 34 patients with unknown cytogenetics and missing beta‐2 microglobulin at baseline were allocated to an 'unclassified' R‐ISS stage at study entry. A comparison of the results from this post hoc analysis is shown in Table 3. The hazard ratios for progression‐free survival remain in favour of isatuximab plus pomalidomide and dexamethasone in all R‐ISS stage subgroups, independently of the R‐ISS allocation method used.

**TABLE 3:** Progression‐free survival based on disease assessment by the independent response committee by original R‐ISS allocation and alternative R‐ISS allocation, intent‐to‐treat population

	Isa‐Pd			Pd				
	*n*	*n* (%) of events	Median (months) (95% CI)	*n*	*n* (%) of events	Median (months) (95% CI)	Hazard ratio vs. Pd (95% CI)	*p*‐value for interaction
All patients	154	73 (47.4)	11.532 (8.936–13.897)	153	89 (58.2)	6.472 (4.468–8.279)	0.596 (0.436–0.814)	–
R‐ISS stage at study entry, original allocation								
Stage I	39	13 (33.3)	14.784 (11.203–NC)	31	17 (54.8)	10.382 (7.754–NC)	0.584 (0.283–1.205)	0.9871
Stage II	99	47 (47.5)	11.499 (7.491–15.211)	98	57 (58.2)	6.472 (4.041–9.528)	0.587 (0.398–0.868)	
Stage III	16	13 (81.3)	2.793 (1.840–9.495)	24	15 (62.5)	1.971 (1.248–3.285)	0.605 (0.280–1.307)	
R‐ISS stage at study entry, alternative allocation								
Stage I	31	11 (35.5)	14.784 (11.203–NC)	20	12 (60.0)	10.382 (5.848–NC)	0.606 (0.261–1.406)	0.9215
Stage II	91	44 (48.4)	11.400 (6.472–15.211)	91	52 (57.1)	7.031 (4.041–9.758)	0.619 (0.412–0.928)	
Stage III	16	13 (81.3)	2.793 (1.840–9.495)	24	15 (62.5)	1.971 (1.248–3.285)	0.605 (0.280–1.307)	
Unclassified	16	5 (31.3)	NC (8.246–NC)	18	10 (55.6)	7.754 (2.595–NC)	0.426 (0.145–1.253)	

Abbreviations: Cl, confidence interval, d, dexamethasone; Isa, isatuximab; NC, not calculable; P, pomalidomide; R‐ISS, revised international staging system.

Phase 3 trials studying patients with relapsed/refractory multiple myeloma since the R‐ISS was developed [2] started reporting survival by R‐ISS stages I‐III subgroups if high‐risk cytogenetics data were available. However, these results are not calculated for the R‐ISS unclassified subgroup. For example, in APOLLO, the Phase 3 study of daratumumab plus pomalidomide and dexamethasone versus pomalidomide and dexamethasone, the median progression‐free survival for R‐ISS stage I was not estimable in the daratumumab arm versus 10.4 months in the control arm, with a hazard ratio (95% confidence interval) of 0.51 (0.24–1.10). The median progression‐free survival for R‐ISS stages II and III were 12.3 months and 2.8 months in the daratumumab arm versus 6.5 and 3.4 months in the control arm, with hazard ratios (95% confidence intervals) of 0.58 (0.39–0.85) and 1.38 (0.62–3.11), respectively [3].

It is important to note that with the original R‐ISS allocation method used in the ICARIA‐MM study, patients with R‐ISS stage III were not inappropriately allocated to either stage II or stage I. Additionally, the number of patients in each unclassifiable cohort in the alternative R‐ISS allocation approach, that is, *n* = 16 for isatuximab plus pomalidomide and dexamethasone and *n* = 18 for the pomalidomide and dexamethasone, is low. Assuming that a truly random population of patients is part of these cohorts, it is also likely that only a significant randomization error could produce a real difference in outcome, such as having all patients in stage I in one cohort and stage III in another. Since this is an unlikely event, the alternative R‐ISS allocation approach then reflects the whole population regardless of R‐ISS stage. Because the conclusion of the trial is that all stages improve outcomes, then the whole population should equally do so. Although the original R‐ISS allocation method used in ICARIA‐MM did not ultimately change the interpretation of the results, in studies where the missing data are larger than it was in ICARIA‐MM, it might be important to avoid using the original R‐ISS allocation approach described in this letter. Finally, the conclusions of ICARIA‐MM results did not change, supporting the use of these different methodologies and the data derived as part of the broader conclusions from this Phase 3, approval‐finding study [4].

## CONFLICT OF INTEREST

Dr. Richardson has received the research funding from Bristol‐Myers Squibb, Celgene, Oncopeptides and Takeda and reports honoraria from Celgene, Janssen, Karyopharm, Oncopeptides, Sanofi and Takeda. Prof. Perrot has received honoraria from Amgen, Bristol‐Myers Squibb/Celgene, Janssen, Sanofi and Takeda. Dr. Takamatsu has received research funding from Bristol‐Myers Squibb and Janssen and reports honoraria from Adaptive Biotechnologies, Bristol‐Myers Squibb, Janssen, Ono and Sanofi.

## AUTHOR CONTRIBUTIONS

Paul G. Richardson and Aurore Perrot were coprimary investigators of the ICARIA‐MM study. Hiroyuki Takamatsu contributed to the analysis and interpretation of data for the work. All authors revised the work for important intellectual content and assume responsibility for data integrity and the decision to submit this manuscript for publication; had full access to the study data; and edited and reviewed manuscript drafts, and approved the final version for submission.

## FUNDING INFORMATION

Sanofi. Qualified researchers can request access to patient‐level data and related study documents including the clinical study report, study protocol with any amendments, blank case report forms, statistical analysis plan, and dataset specifications. Patient‐level data will be anonymised, and study documents will be redacted to protect the privacy of trial participants. Further details on Sanofi’s data‐sharing criteria, eligible studies, and process for requesting access are at: https://www.clinicalstudydatarequest.com.
